# Sorafenib and nitazoxanide disrupt mitochondrial function and inhibit regrowth capacity in three-dimensional models of hepatocellular and colorectal carcinoma

**DOI:** 10.1038/s41598-022-12519-4

**Published:** 2022-05-27

**Authors:** Frida Ek, Kristin Blom, Tove Selvin, Jakob Rudfeldt, Claes Andersson, Wojciech Senkowski, Christian Brechot, Peter Nygren, Rolf Larsson, Malin Jarvius, Mårten Fryknäs

**Affiliations:** 1 Department of Medical Sciences, Cancer Pharmacology and Computational Medicine, Uppsala University Hospital, Uppsala University, 751 85 Uppsala, Sweden; 2 Biotech Research & Innovation Centre, Novo Nordisk Foundation Center for Stem Cell Biology, University of Copenhagen, 2200 Copenhagen N, Denmark; 3 University of South Florida and Romark L.C., Tampa Florida, USA; 4 Department of Immunology, Genetics and Pathology, Uppsala University, 751 85 Uppsala, Sweden; 5 Present Address: Department of Pharmaceutical Biosciences and Science for Life Laboratory, Uppsala University, Box 591, 751 24 Uppsala, Sweden

**Keywords:** Cancer, Cancer metabolism, Cancer models, Cancer therapy, Gastrointestinal cancer

## Abstract

Quiescent cancer cells in malignant tumors can withstand cell-cycle active treatment and cause cancer spread and recurrence. Three-dimensional (3D) cancer cell models have led to the identification of oxidative phosphorylation (OXPHOS) as a context-dependent vulnerability. The limited treatment options for advanced hepatocellular carcinoma (HCC) and colorectal carcinoma (CRC) metastatic to the liver include the multikinase inhibitors sorafenib and regorafenib. Off-target effects of sorafenib and regorafenib are related to OXPHOS inhibition; however the importance of this feature to the effect on tumor cells has not been investigated in 3D models. We began by assessing global transcriptional responses in monolayer cell cultures, then moved on to multicellular tumor spheroids (MCTS) and tumoroids generated from a CRC patient. Cells were treated with chemotherapeutics, kinase inhibitors, and the OXPHOS inhibitors. Cells grown in 3D cultures were sensitive to the OXPHOS inhibitor nitazoxanide, sorafenib, and regorafenib and resistant to other multikinase inhibitors and chemotherapeutic drugs. Furthermore, nitazoxanide and sorafenib reduced viability, regrowth potential and inhibited mitochondrial membrane potential in an additive manner at clinically relevant concentrations. This study demonstrates that the OXPHOS inhibition caused by sorafenib and regorafenib parallels 3D activity and can be further investigated for new combination strategies.

## Introduction

Prior research demonstrated that cancer cells primarily rely on glycolysis for survival and proliferation, known as the Warburg effect^1^. However, it is now clear that cells, under certain conditions, require OXPHOS for their metabolic demands and that enhanced OXPHOS is a characteristic of cancer stem cells and cancer cells with inherent or acquired drug resistance^2–4^. Furthermore, oxygen and nutritional gradients arise at radial distances from blood vessels in solid tumors. This allows for fast cell proliferation near blood vessels. In contrast, more distant cells suffer from hypoxia, starvation, and dropping pH, all of which drive cells to a quiescent and OXPHOS-dependent state^5,6^. Quiescent cells are non-proliferating and thus insensitive to cell-cycle active chemotherapy, allowing for disease relapse and metastasis^7^. Therefore, these cells are high-priority targets when developing new cancer treatment strategies. Since the hypoxic and nutrient-deprived cells cannot rely solely on glycolysis to meet their energy demands, drugs that impair OXPHOS seem to be promising treatment alternatives^2,4,8^.

Quiescent OXPHOS-dependent cancer cells can be modeled using three-dimensional (3D) cell cultures in which hypoxia, glucose deprivation, and low pH mimic the avascular tumor microenvironment^9^. A 3D model of moderate complexity suitable for high-throughput testing is the multicellular tumor spheroid (MCTS) model^10^. MCTS consist of cells from immortalized cell lines that are allowed to self-assemble into spheroids. Cells growing in the MCTS periphery, with access to oxygen and nutrients, can proliferate. In the center of the MCTS, the conditions are hypoxic and the pH acidic, causing cells to be slowly-proliferating, quiescent, or even necrotic^11^. If a limited amount of cell medium is used and not replaced during the culture period, a larger fraction of the cells will become quiescent^12^.

The tumoroid model established from primary cultures of patient tumor cells is more complex and, potentially, more clinically relevant. They develop tumor-like structures by self-assembly, have a high cellular heterogeneity, and often resembles the original tumor both genetically and phenotypically^13–15^. Tumoroids constitute a unique possibility to study clinically relevant treatment efficacy in vitro, predict drug sensitivity in vivo^15^ and guide personalized cancer therapy^13,15^.

Screening efforts using 3D cell models have revealed that mitochondrial OXPHOS is a vulnerability that can be exploited therapeutically^2,7,16,17^. Several anthelmintic drugs, including nitazoxanide and niclosamide, have been identified in phenotypic MCTS based screens as repurposing candidates^12,18,19^. Nitazoxanide and niclosamide inhibit OXPHOS via mitochondrial uncoupling, and both have substantial effects on quiescent MCTS^12,20^. Several other mitochondrial inhibitors have also been developed^12^, including the mitochondrial complex I inhibitor IACS-010759 currently in clinical trials^21^.

The multikinase inhibitors sorafenib and its close structural analog regorafenib (see Supplementary Table 1 for comparison) both have OXPHOS inhibitory properties in vitro^22–26^. This off-target effect may be relevant to the clinical activity of sorafenib and regorafenib, and such insight may be valuable for treatment optimization. Thus, we investigated whether OXPHOS inhibition by sorafenib and regorafenib, alone or in combination with nitazoxanide, is effective in 3D models of hepatocellular and colorectal carcinoma.

## Results

### Global gene expression and galactose experiments indicate similarity between sorafenib and OXPHOS inhibitors

**Figure 1:**
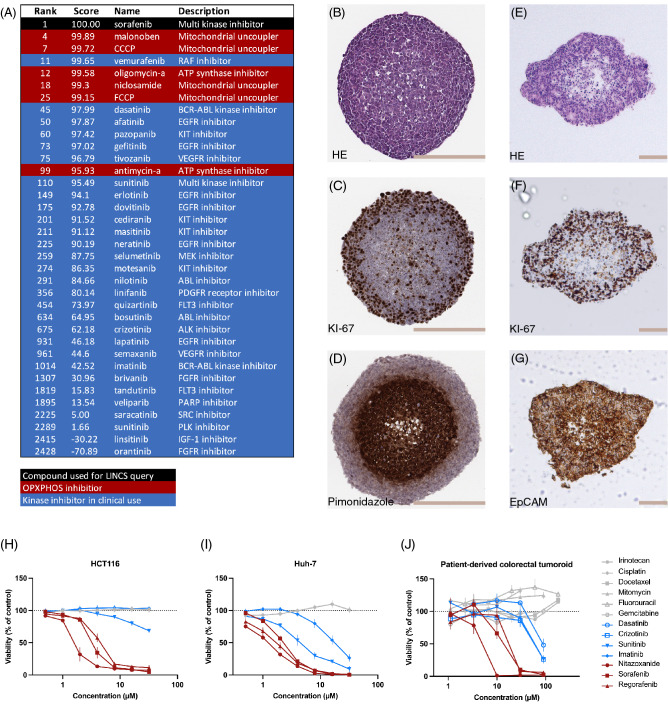
Sorafenib response in 2D and 3D models compared to OXPHOS inhibitors, kinase inhibitors, and cytotoxic compounds. **(A)** Global gene expression-based response analysis (L1000, LINCS database). **(B–D)** IHC staining of HCT116 MCTS with hematoxylin–eosin **(B)**, ki-67 **(C)**, hypoxia marker pimonidazole **(D)**. Scale bars = 200 μm. **(E–G)** IHC staining of tumoroids in hematoxylin–eosin **(E)**, proliferation marker ki-67 **(F)**, and epithelial marker EpCam **(G)**. Scale bars = 100 μm. (**H,I**) Viability, plotted as mean ± SD, after treatment with six compounds for 72 h, measured using ATP-assay in HCT116 MCTS **(H)** n = 4 (quadruplicate wells), Coefficient of variation CV(controls) = 5% and Huh-7 MCTS **(I)** n = 3 (quadruplicate wells), CV(controls) = 5%. **(J)** Tumoroid viability, after treatment with 12 compounds for 72 h, was measured using the ATP-assay. The graph plots mean ± SD, n = 1 (duplicate wells for all compounds but nitazoxanide which was run in quadruplicates wells). CV(controls) = 8%.

It has been clearly shown and well-documented that the multikinase inhibitors sorafenib and regorafenib have off-target effects causing OXPHOS inhibition in monolayer (2D) cultures^22,23,26^ most likely caused by mitochondrial uncoupling^25^. Here we first hypothesized that this mitochondrial effect could elicit an immediate response at the global gene expression level, which would imply that induction of mitochondrial dysfunction plays a role in the mode of action. We used the LINCS database^27^, a publicly available resource suitable for compound characterization, to explore global effects on gene expression after sorafenib exposure in 2D cultures. LINCS enables transcriptional response comparisons for more than 3000 compounds. After exposure to sorafenib, the global transcription profiles generated from nine different cell lines were, in aggregate, more similar to those of other OXPHOS-inhibitors (e.g., malonoben, niclosamide, CCCP, etc.) than other clinically used kinase inhibitors (Fig. 1A). Thus, this agrees with previous reports of sorafenib-induced mitochondrial dysfunction and indicates that it may be an integral part of the mechanism of action^12,25,28^. To further investigate the possible parallels between the antiparasitic uncoupling drugs (i.e., niclosamide and nitazoxanide) and the multikinase inhibitors sorafenib and regorafenib, monolayer (2D) experiments were performed in medium containing either glucose or galactose (supplementary Fig. 1). Growth in galactose-containing medium prevents glycolytic metabolism, forcing cells to rely solely on oxidative phosphorylation for ATP generation^29^. The 24-h ATP levels demonstrate that niclosamide and nitazoxanide are considerably more active under conditions when functional OXPHOS is required (i.e., galactose containing medium). Sorafenib, regorafenib, and the positive controls CCCP (uncoupler) and oligomycin (ATPase inhibitor) cause similar responses to anti parasitic compounds. On the other hand, the multikinase inhibitor sunitinib and the cytotoxic compounds irinotecan and oxaliplatin are not more effective when grown in galactose as compared to the response in glucose counting medium. Similar results were obtained in the HCC cell line Huh-7 and CRC cell line HCT116 (supplementary Fig. 1).

### Cancer cells in different types of 3D models

We and others have previously shown that well-functioning OXPHOS is critically important in hypoxic and metabolically stressed cancer cells^2,7,12,16,17,20,21^. Here, two types of 3D models, MCTSs and tumoroids from a patient, were used to test the treatment effects after compound exposure. MCTS were generated from Huh-7 and the CRC cell line HCT116. Unlike most other spheroid-based approaches, the current method did not involve any medium changes throughout the spheroid growing phase. In terms of glucose content and pH, we could better simulate circumstances found in dormant tumor areas in vivo^12^. The center of HCT116 MCTSs had a hypoxic interior and structural changes consistent with necrotic core and only a rim of proliferating (i.e., KI-67 positive) cells in the periphery after seven days of growth (Fig. 1B–D). CRC-tumoroids from a patient were more heterogeneous and retained a more in vivo reminiscent 3D organization (Fig. 1E–G). The low density of cells in the core of tumoroids indicates central necrosis (Fig. 1E). They also developed a low proliferation center and a proliferating peripheral zone (Fig. 1F). Epithelial marker EpCam was positive throughout the whole tumoroid (Fig. 1G), indicating that it consisted of colorectal cancer cells of epithelial origin.

### Sorafenib, regorafenib and nitazoxanide are active against cells in 3D models

We tested a panel of compounds in the MCTS models, including previously reported OXPHOS inhibitors (i.e., nitazoxanide and the multikinase inhibitors sorafenib and regorafenib) and compounds without prior documentation on OXPHOS inhibitory capacity (i.e., the multikinase inhibitors sunitinib and imatinib and the cytotoxic compound irinotecan). In the MCTS models, compounds previously documented as OXPHOS inhibitors were dramatically more effective (Fig. 1H,I). This response is specific to the conditions generated in 3D cultures as the same pattern was not found in monolayer cell cultures of HCT116 and Huh-7 (supplementary Fig. 2 and supplementary Tables 4 and 5). When tested in the colon cancer tumoroid model, a similar pattern of sensitivity to nitazoxanide, sorafenib, and regorafenib was observed, whereas other kinase inhibitors (i.e., sunitinib, imatinib, dasatinib, and crizotinib), and cytotoxic drugs (i.e., irinotecan, cisplatin, docetaxel, mitomycin, fluorouracil, and gemcitabine) remained ineffective (Fig. 1J and supplementary Tables 2 and 3). 

### Sorafenib, regorafenib, and nitazoxanide disrupt mitochondrial function in 3D

**Figure 2:**
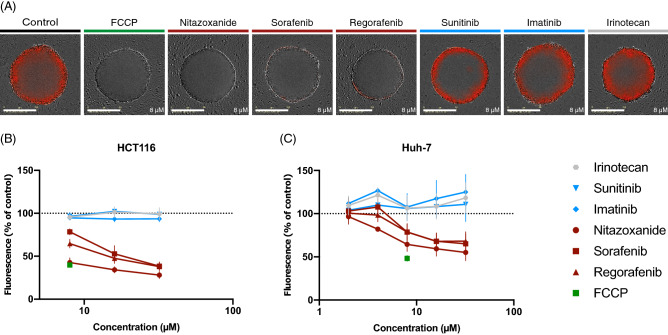
Disruption of mitochondrial function in MCTS. The JC-1 assay was used to visualize alterations in mitochondrial membrane potential (Δψm) utilized as a marker for mitochondrial function. A, Images of HCT116 MCTS treated with seven compounds for 2 h (scale bar = 400 µm). **(A–C)** Mitochondrial membrane potential (Δψm) was decreased by nitazoxanide, sorafenib, regorafenib, and positive control FCCP in both Huh-7 MCTS. Huh-7 treated with nitazoxanide, sorafenib, regorafenib, sunitinib, imatinib, irinotecan: n = 4 (quadruplicate wells) for 8–32 µM and n = 2 (quadruplicate wells) for 2–4 µM, FCCP: n = 2 (quadruplicate wells), CV(controls) = 6.5–10.6%. HCT116: n = 3 (quadruplicate wells), CV(controls) = 7.3–12.5%). Δψm was unaffected by sunitinib, imatinib, and irinotecan, compared to control in both cell lines. **(B,C)** Graphs depicting Δψm as % red fluorescence compared to control (mean ± SEM).

To investigate if mitochondrial membrane potential (Δψm)-disruption can be demonstrated in 3D cultures, we performed the JC-1 assay directly on MCTS formed from Huh-7 and HCT116 cells (Fig. 2). The MCTS were treated for 2 h with compounds that have reported OXPHOS-activity (i.e., nitazoxanide, sorafenib, regorafenib, and the positive control FCCP) and compounds that do not affect mitochondria (i.e., sunitinib, imatinib, and irinotecan). Mitochondrial membrane potential (Δψm) was decreased in MCTS after exposure to nitazoxanide, sorafenib, regorafenib, and positive control FCCP. At the same time, sunitinib, imatinib, and irinotecan did not affect mitochondrial membrane potential, compared to untreated control (Fig. 2, supplementary Fig. 3 and supplementary Table 6). Concentrations that affect the mitochondrial potential (Fig. 2B,C) are comparable to the concentrations that affect viability in 3D (Fig. 1D,H,I).

### Sorafenib affects colony expansion

**Figure 3:**
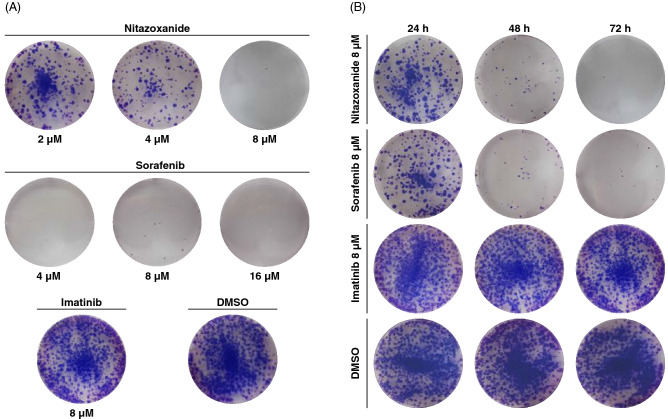
Sorafenib affects colony expansion, and duration of treatment is critical. (**A**) using clonogenic assay, HCT116 MCTS decreased their colony expansion capacity after sorafenib and nitazoxanide treatment for 72 h, while imatinib and DMSO did not. (**B**) Treatment with concentrations of 8 µM for > 24 h was necessary to ensure low re-growth potential using sorafenib and nitazoxanide. One representative well from one experiment shown, numeric results on replicate experiments in supplementary table 3, n = 3 (triplicate wells) for all treatments but imatinib treatment (n = 2 (triplicate wells).

Clonogenic assay was performed after treatment of MCTS, formed from HCT116 cells, to corroborate the ATP measurements (Fig. 1H) with an orthogonal assay. After treatment, the MCTS were disintegrated enzymatically and re-seeded at low density to allow regrowth and colony formation. When MCTs are grown according to this method, almost the entire MCTS consists of quiescent cells after 7 days of growth in a depleted medium^12,30^. This experiment aimed to investigate if sorafenib can eradicate quiescent cancer cells as previously demonstrated for nitazoxanide^12^. Both nitazoxanide and sorafenib inhibited colony regrowth potential in HCT116 MCTS, sorafenib already at 4 mM. Imatinib, irinotecan, and DMSO did not affect colony expansion in HCT116 MCTS (Fig. 3A and supplementary Table 8).

### Importance of exposure duration

To evaluate possible treatment regimes, exposure-effect relationships were established. When exposed for 72 h, 3D grown cells were sensitive to sorafenib (Figs. 1H–J and 3A). However, disruption of mitochondrial function occurs already two hours post-treatment (Fig. 2). To shed light on treatment duration requirement, we performed a clonogenic regrowth assay after 24, 48, and 72 h of drug exposure (Fig. 3B). Colony formation inhibition correlated to exposure time for nitazoxanide and sorafenib, while exposure duration of imatinib and irinotecan did not affect colony regrowth. This supports the notion that sustained treatment is crucial and reproduces previous results for nitazoxanide^12^. Colony counts and statistical analysis from three replicates are shown in supplementary Fig. 5 and supplementary table 8.

### Combining sorafenib and nitazoxanide results in additive effects on viability, mitochondrial function, and colony expansion

**Figure 4:**
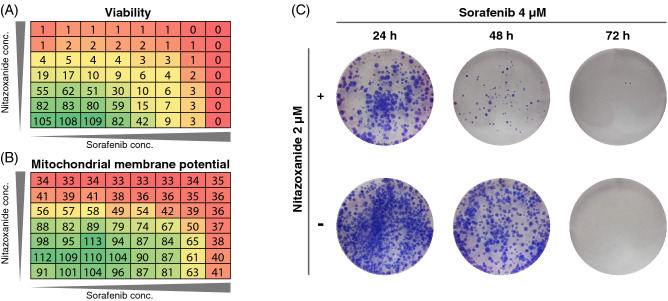
Combining sorafenib and nitazoxanide generates additive effects in HCT116 MCTS. A-B, MCTS were treated with nitazoxanide (1–32 µM), and sorafenib (0.5–32 µM) in a combination matrix for 72 h resulting in additive effects in both viability using ATP-assay analyzed with MacSynergy tool (**A**, n = 3 (quadruplicate wells), survival % of control) and Δψm (**B**, n = 2 (quadruplicate wells), fluorescence % of control). (**C**) MCTS treated with sorafenib (4 µM) in combination with nitazoxanide (2 µM) for 48 h and 72 h decreased the colony expansion capacity (n = 2 (triplicate wells), additional data in supplementary Table 3).

Since sorafenib and regorafenib have dose-limiting side effects, combination therapy with another mitochondrial inhibitor might improve the therapeutic potential. Therefore, sorafenib and nitazoxanide were combined to evaluate whether the efficacy, disruption of mitochondrial function, and colony expansion potential indicate additive effects. When sorafenib and nitazoxanide were added to MCTS in a combination matrix between 0.5 and 32 mM, additive effects were visible in ATP-assay (Fig. 4A), mitochondrial function (Fig. 4B, supplementary Fig. 4 and supplementary Table 7), and colony expansion (Fig. 4C and supplementary Fig. 5 and supplementary Table 8). Importantly, we have used concentrations similar to what can be clinically achieved for sorafenib (C_max_ 4.3 µM^23^), regorafenib (C_max_ 8.1 µM^23^), and nitazoxanide (6 µM^31^). A synergy analysis was also performed using MacSynergy^32^ where additive effects were demonstrated (data not shown). These results indicate that sorafenib and nitazoxanide have additive effects on quiescent cancer cells and should be further investigated as a promising combination treatment.

## Discussion

We propose that the previously described mitochondrial effects of sorafenib and regorafenib at clinically relevant concentrations translate into potent activity against quiescent cancer cells in 3D models. This property can be used as a basis for combination therapy with less toxic OXPHOS inhibitors.

Over the last few decades, the role of functioning OXPHOS in cancer cells has become increasingly evident, and direct links between tumorigenicity, stemness, and vital mitochondria have been demonstrated^8^. For example, cancer cells that lack mtDNA (Rho-0) have been utilized to establish the association between functional OXPHOS and retained tumorigenicity^33^. Metformin, used for the treatment of type 2 diabetes, can act as an inhibitor of complex I and specifically induces metabolic stress and apoptosis in cancer stem cells, causing a decrease in tumorigenic cells in vivo^34^. Metformin is currently undergoing numerous clinical trials for a variety of cancers. The two complex I inhibitors, IACS-010759 and BAY87-2243, have entered clinical development, as well as the mitochondrial inhibitor ME-344^21,35,36^.

The importance of lipophilicity to penetrate cancer parenchyma has been previously described, and a connection between higher logP value and increased efficacy in 3D models has been established^37^. Substances with effect against quiescent cells in this study all have relatively high lipophilicity (XlogP ≥ 2, see supplementary Table 1). In contrast, conventional cytostatic compounds like 5-fluorouracil and gemcitabine have considerably lower lipophilicity (XlogP −0.9 and −1.5, respectively, supplementary Table 1). However, both irinotecan and docetaxel have higher XlogP. Thus, a high XlogP can be considered required but not sufficient for activity in 3D cultures.

OXPHOS inhibition as a therapeutic strategy has been clinically evaluated, as exemplified by 2,4-dinitrophenol (2,4-DNP). The ammunition-ingredient 2,4-DNP induced weight loss after reports of exposure-related poisoning in factories during the first world war. Within a year after the first published clinical study in the 1930s, over 100,000 people had taken 2,4-DNP, and the results in terms of weight loss were impressive. However, there was a wide range of reported side effects including fatally increased body temperature, which led to the withdrawal of 2,4-DNP^38–40^. In this context, it may be worth noting that fever is a well-documented side effect of nitazoxanide, sorafenib, and regorafenib treatment. Furthermore, in patients with HCC treated with sorafenib, fever has been described as an independent predictor of good therapy response^41^. Naturally, the severity of side effects that may be tolerated varies depending on whether the aim is weight loss or cancer treatment.

We show that the global transcription profiles induced by sorafenib are similar to those elicited by OXPHOS inhibitors, highlighting the role of mitochondrial uncoupling in sorafenib's mechanism of action. When sorafenib and regorafenib were added to cells grown in galactose-containing media, ATP was depleted at clinically relevant concentrations, indicative of OXPHOS inhibition. This is in line with previous studies^12,25,28^ and similar to the effects we found following exposure to the antiparasitic uncouplers nitazoxanide and niclosamide^12,30^. Sorafenib and regorafenib diminish mitochondrial membrane potential, while the other kinase inhibitors did not. A clear difference in sensitivity in 3D cancer models was also observed in two cell lines and patient-derived CRC-tumoroids in which sorafenib and regorafenib were more effective against cancer cells in 3D cell cultures. The concentrations of sorafenib and regorafenib that inhibit cancer cell regrowth in 3D models match the concentrations that disrupt mitochondrial function. Sorafenib had a powerful effect on colony expansion, even more potent than nitazoxanide, corroborating the efficacy of sorafenib on cancer cells in 3D-cell cultures.

Hypoxia generated by aberrant microvasculature and unregulated cell proliferation is frequent in HCC patients^42,43^. Hypoxia enhances tumor aggressiveness and chemoresistance by interfering with many biological processes in HCC^43–45^. Moreover, in Transarterial chemoembolization (TACE), which is considered the first-line treatment for patients with intermediate stage HCC^46^ hypoxia is inherent to the procedure and thought to limit the efficacy of the intervention ^47,48^. Thus, the findings presented here may be of value for developing new TACE treatment protocols.

As broad tyrosine kinase inhibitors, both sorafenib and regorafenib have VEGF inhibitory actions, inhibiting angiogenesis and causing tumor hypoxia and nutrition deprivation. It seems reasonable to assume that increasing hypoxia and nutritional restriction will enhance tumor sensitivity to OXPHOS inhibition. Resistance to sorafenib is associated with increased OXPHOS-capacity, further emphasizing the importance of mitochondrial uncoupling in the mechanism of action of sorafenib^49^. Sorafenib and regorafenib both have dose-limiting side effects. Therefore, it could be beneficial to combine the treatment of sorafenib or regorafenib with a well-tolerated OXPHOS inhibitor like nitazoxanide to ensure prolonged and constant treatment pressure on the cancer cells. Nitazoxanide reached plasma concentration > 6 µM after a single oral dose of 500 mg^50^ and has been administered to over 75 million people with gastrointestinal infections without major drug-related safety issues^51^. Sorafenib and nitazoxanide both directly affect mitochondria through uncoupling^12,25,28,52^, which likely explains their additive effect in our 3D models. Although this study is limited to in vitro experiments, it is worth noting that sorafenib and niclosamide have previously shown good combination effects in a rodent HCC model^53^. In a clinical setting, if the OXPHOS inhibition elicited by nitazoxanide and sorafenib is additive, this drug combination may benefit patients. Given the structural (supplementary Table 1) and phenotypic similarities demonstrated here (supplementary Fig. 1) and in previous reports^22,23^, we propose that regorafenib might be considered for combination therapy using the same logic. Therefore, we suggest that nitazoxanide and sorafenib/regorafenib combinations should be further investigated to treat HCC and recurrent or metastasized CRC. In conclusion, our results show that mitochondrial off-target effects parallels sorafenib and regorafenib activity in 3D models, which might have therapeutic implications.

## Materials and methods

### Cell culture

The human HCC cell line Huh-7 (CVCL_0336), kindly provided by Dr. Nicolas Moniaux, Inserm, Paris, France, was cultured in high glucose DMEM (D6546, Sigma-Aldrich) supplemented with 10% HI FBS (F9665, Sigma-Aldrich) 2 mM glutamine (G7513, Sigma-Aldrich) and Penicillin (100U/ml)/Streptomycin (100 μg/ml) (P4333, Sigma-Aldrich). HCT116 (CVCL_0291, ATCC) and the human CRC cell line HCT116-GFP (Anticancer) were cultured in McCoy’s 5A medium (M8403, Sigma-Aldrich) supplemented with 10% HI FBS (F9665, Sigma-Aldrich) 2 mM glutamine (G7513, Sigma-Aldrich) and Penicillin (100 U/ml)/Streptomycin (100 μg/ml) (P4333, Sigma-Aldrich). The cell lines were kept at 37 °C in 5% CO_2_, subcultivated, and viability controlled twice a week. Experiments in galactose was performed as described^29^. Briefly, DMEM (11966-025, Invitrogen) free of glucose was supplemented with 10 mM galactose (G5388, Sigma-Aldrich), 2 mM glutamine (G7513, Sigma-Aldrich) plus 4 mM before to supplementation to generate a final concentration of 6 mM), 10% HI FBS (F9665, Sigma-Aldrich), 1 mM sodium pyruvate (P5280, Sigma-Aldrich), and Penicillin (100 U/ml) and Streptomycin (100 μg/ml) (P4333, Sigma-Aldrich). High-glucose media. DMEM (11995-065, Invitrogen) containing 25 mM glucose and 1 mM sodium pyruvate. Supplemented with 10% HI FBS (F9665, Sigma-Aldrich) and Penicillin (100 U/ml) and Streptomycin (100 μg/ml) (P4333, Sigma-Aldrich). HCT116-GFP and Huh-7 were grown in galactose-containing media and high glucose media respectively. The cells were kept in 5% CO_2_ at 37 °C and were cultured for at least six passages before experiments were performed. All cell lines were checked for mycoplasma infection and sent to Eurofins Genomics (Ebersberg, Germany) for cell line authentication using DNA and short tandem repeat profiles.

### Measurement of cellular ATP content

Cells were seeded in 384-well plates (142761, Thermo Fisher Scientific) at a density of 5000 cells/well (HCT116-GFP) and 4000 cells/well (Huh-7) in 50 µl medium. Drugs were added 24 h after seeding using an Echo 550 Liquid handler (Beckman Coulter). ATP Content was measured after 24 h of drug treatment using CellTiter-Glo® 2.0 Cell Viability Assay (G9242, Promega) according to the manufacturer’s recommendations. Luminescence was measured using a microplate reader (FLUOstar Omega, BMG LabTech).

### Establishment of MCTS from cell lines

Cells were seeded (5000 cells/50 µl/well) into Ultra-low attachment 384-well spheroid microplates (3830, Corning) and centrifuged at 200*g* for 5 min. MCTS self-assembled over seven days without interference or medium change at 37 °C in 5% CO_2_. A Breathe-Easy sealing membrane (Z380059, Sigma-Aldrich) was used to minimize evaporation and prevent contamination and sample spillage.

### Patient cell preparation

Sample preparation of patient tumor cells has previously been described^54,55^. In short, colorectal carcinoma samples were collected in sterile transport media, minced, and enzymatically treated to obtain a cell suspension containing single cells and small aggregates. Successful isolation resulted in a cell suspension with a tumor cell count and viability greater than 70%, respectively. The cells were then cryopreserved in freezing media (HI-FBS containing 10% DMSO) and placed in a -150 °C freezer for long-term storage.

### Establishment of tumoroids from patient cells

Colorectal carcinoma cells (0,5*10^6^ cells/ml) were suspended in ice-cold Cultrex® Basement Membrane Extract (BME) gel (10 mg/ml in PBS (D8537, Sigma-Aldrich) (3533-005-02, Bio-Techne) and seeded in 20 μl droplets in 6-well plates (Nunclon Delta 140685, Thermo Fisher Scientific) at a density of 10 droplets per well. The gel droplets were solidified for 30 min at 37 °C in 5% CO_2_ before the addition of 3 ml per well of Advanced DMEM/F-12 medium (12634010, Gibco) supplemented with 10 ml B-27 (50×) (11530536, Gibco), 5 ml N1 (100×) (N6530, Sigma-Aldrich), 10 µg basic fibroblast growth factor (hbFGF) (HBFGF-RO, Sigma-Aldrich), 50 µl epidermal growth factor (EGF) (E9644, Sigma-Aldrich), 10 mM HEPES (H0887, Sigma-Aldrich), Penicillin (100 U/ml)/Streptomycin (100 μg/ml) (P0781, Sigma-Aldrich), 81,6 mg *N*-acetyl-l-cystein (A7250, Sigma-Aldrich), 217.2 mg Ala-Gin (A8185, Sigma-Aldrich) and 10 µM Rock II (Y0503, Sigma-Aldrich).

Tumoroid droplets were grown at 37 °C in 5% CO_2_ for 21 days with medium change every 3–4 days. After 21 days, 2 ml/well of medium was removed, and the plates were placed at 4 °C for 30 min to loosen up the gel. All gel droplets were moved to a 50 ml Falcon tube and centrifuged at 200*g* for 10 min. The tumoroid cell pellet was washed with PBS 2–3 times with centrifugation at 200*g* for 10 min to remove the gel. A sample of the tumoroids was collected for immunohistochemistry staining (see “Immunohistochemistry” below), and the remaining cells were treated with Accumax™ (A7089, Sigma-Aldrich) to separate the structures further. Finally, the tumoroid cells were suspended in ice-cold Cultrex® BME gel (10 mg/ml in PBS (D8537, Sigma-Aldrich) (3533-005-02, Bio-Techne)) and seeded at a density of 5000 cells/well in 5 μl droplets in a 384-well plate (Nunc™ 384-well polystyrene black (142761, Thermo Fisher Scientific)) with an additional 40 µl medium (same medium as above). The tumoroids were cultured at 37 °C in 5% CO_2_ for 21 days with medium change every 3–4 days before being subjected to drug treatment using an Echo 550 Liquid handler (Beckman Coulter). After 72 h viability of the tumoroids was analyzed using CellTiter-Glo® 3D Cell Viability Assay (G9682, Promega) according to the manufacturer’s recommendations. Mutation analysis using TruSight Oncology 500 (TSO500; Illumina, San Diego, CA), as previously described^56^, for a wide range of oncogenes and tumor suppressor genes (n = 523, e.g., TP53 mutation, APC mutation, MYC amplification, etc.), from both primary and secondary organoid cultures, agree with the original diagnosis (colorectal cancer) and indicate that no or only minor genetic changes were induced during the organoid culture period.

### Viability assay

Drugs dissolved in DMSO (D5879, Honeywell/Fisher Scientific) or pharmacy stock solution (cisplatin) were added to 384-well plates with either MCTS, tumoroids or monolayer culture using an Echo 550 Liquid Handler (Beckman Coulter), and the plates were then incubated for 72 h at 37 °C in 5% CO_2_ before analyzing viability using CellTiter-Glo® 3D Cell Viability Assay (G9682, Promega) or CellTiter-Glo® 2.0 Cell Viability Assay (G9242, Promega) according to the manufacturer’s recommendations. Luminescence was measured using a microplate reader (FLUOstar Omega, BMG LabTech), and the following equation calculated the survival index (SI%):

$$SI\% \, = \,100\, \times \,f\left( {treated} \right){-}f \, \left( {average \, blank} \right) \, / \, f \, \left( {average \, DMSO \, control} \right){-}f \, \left( {average \, blank} \right)$$

Here, f denoted the luminescence. For information on compounds used see supplementary Table 1.

### LINCS analysis

Clue touchstone (Broad institute) version 1.1.1.2. L1000 is a Library of Integrated Network-based Cellular Signatures (LINCS) where transcriptomic profiles of pharmacologic screening data across nine cell lines are generated and matched to perturbations with similar transcriptomic profiles. Touchstone is a tool in which ~ 3000 annotated compounds with known activities and targets are matched to the compound-induced transcriptomic profile of choice. Sorafenib was used as a seed compound for the similarity search.

### Immunohistochemistry

Fixation and paraffin embedding of the MCTS and the tumoroids were performed in glass vials at room temperature (RT) if not stated otherwise. First, the medium was replaced with 4% formaldehyde (2525459, Sigma-Aldrich), followed by incubation overnight. The MCTS/tumoroids were washed with tap water for 10 min, aspirated, and incubated with Erythrosine B (200964, Sigma-Aldrich) 1.9 mM diluted in 66% acetic acid (A6283, Sigma-Aldrich) for 3 min. The solution was replaced by 70% EtOH for 45–60 min, followed by 95% EtOH for 45–60 min, 100% EtOH for 45–60 min, and xylene for 45–60 min. The xylene was aspirated and replaced with paraffin (65 °C) for 15 min at 65 °C; then the tubes were inverted 5 times before incubating overnight at 65 °C. The next day the paraffin was poured into molds and, when solid sliced in 3 µm slices using a microtome and mounted on glass slides. For the hematoxylin–eosin staining, the slides were deparaffinized using xylene (3 times 3 min); EtOH 100% (2 times 1 min); EtOH 95% (2 times 1 min), and ddH2O for 1 min. The slides were incubated with Mayer’s hematoxylin for 10 min, washed in tap water (30 s), lithium carbonate (30 s), and tap water (30 s) before incubation with eosin (3 min).

After the staining, the slides were dehydrated using EtOH 95% (2 times 1 min), EtOH 100% (2 times 1 min), and xylene (3 times 3 min). For the KI67 and Pimonidazole stainings, the slides were pretreated with EnVision FLEX Target Retrieval Solution, Low pH (K800521-2, Agilent) in a PT Link at 97 °C for 20 min. The slides stained with EpCam were pretreated with EnVision FLEX Target Retrieval Solution, High pH (K800421-2, Agilent) in a PT Link at 97 °C for 20 min. For all antibody stainings, EnVision FLEX, High pH (Link) (K800021-1, Agilent) visualization system was used according to the manufacturer’s recommendations. Briefly, the slides were blocked with peroxidase blocking reagent for 5 min; incubated with primary antibodies EpCam (Sc-25308, Santa Cruz) at 1:500 dilution for 10 min, KI67 (M7240, Dako) at 1:50 dilution for 20 min or Pimonidazole (MAB-1, Hypoxyprobe) at 1:50 dilution for 20 min; secondary HRP-conjugated antibody for 20 min and DAB for 10 min in an Autostainer Link 48 (Agilent). The slides were washed with Tris-buffered saline (TBS) between all steps and stained with Mayer’s hematoxylin for 3 min.

### JC-1 3D

MCTS (see the establishment of MCTS from cell lines above) were, after aspiration of the medium, incubated with 35 µl 3,58 µg/ml JC-1 (T4069, Sigma-Aldrich) in PBS for 30 min at 37 °C in 5% CO_2_. MCTS were then washed with 50 µl PBS/well twice before 50 µl medium was added to each well, followed by drugs using an Echo 550 Liquid Handler. The plates were centrifuged at 200*g* for 5 min, and the MCTS incubated with the drugs for 2 h before scanning in IncuCyte S3 Live-Cell Analysis System (Essen Bioscience). The spheroid module Basic Analyzer was used in 10 × magnification, and red image mean uncorrected was plotted. Fluorescence as % of control was calculated in MS Excel for all individual wells.

### Clonogenic assay

HCT116-GFP-MCTS (see above) were treated using Echo Liquid Handler (Beckman Coulter) on day seven and incubated with the drugs for 24, 48, and 72 h at 37 °C in 5% CO_2_. The MCTS were dispersed using Accumax™ (50 µl/well, 30 min, 37 °C in 5%) (A7089, Sigma-Aldrich) and meticulous pipetting until a single cell solution was obtained. The plates were centrifuged for 5 min at 200*g*, the Accumax™ was aspirated using Elx405 Select Deep Well Washer (BioTek), and 50 µl new medium was added per well. From each well 10 µl was transferred to a well of a 6-well plate (140685, Thermo Fisher Scientific) containing 3 ml medium. The cells were left to grow into colonies for ten days at 37 °C in 5% CO_2_ before briefly washing the colonies with PBS (D8537, Sigma-Aldrich), fixating with methanol (A456-1, Fisher Scientific), and staining for 30 min at room temperature with 5% Giemsa (HX263489, Merck) solution in PBS. The Giemsa solution was washed off with water. The colonies were manually counted up to 200 before counting was stopped. The plates were scanned using a Canon Image Runner Advance C5535i printer (Canon) at 600 × 600 dpi resolution.

### Statistical analysis

Raw data was analyzed in MS Excel and GraphPad Prism (version 8.1.1 for macOS). Concentration–response-graphs from viability analysis was analyzed using IC50 calculations in GraphPad Prism. Raw data from the mitochondrial membrane potential-assay and colony regrowth potential assay were analyzed using One-way ANOVA followed by Dunnett’s multiple comparisons. Combination effects on viability and mitochondrial membrane potential were analyzed using MacSynergy tool^32^.

### Ethical considerations

All methods were performed in accordance with the relevant guidelines and regulations, and patient material was handled according to approval by the local ethical committee (Etikprövningsnämnden, Dnr 2007/237). Patient tumor sampling was approved by the local ethical committee (Etikprövningsnämnden, Dnr 2007/237). All patients provided written informed consent prior to sampling.

## Supplementary Information

Supplementary Information.

## Data Availability

Additional data to support the findings in this study are available as supplementary material or may be requested from the corresponding author.
