# Supplementary material for: Sorafenib and nitazoxanide disrupt mitochondrial function and inhibit regrowth capacity in three-dimensional models of hepatocellular and colorectal carcinoma
Source: Sci Rep. 2022 May 27;12:8943. doi: 10.1038/s41598-022-12519-4 (PMC9142582; doi:10.1038/s41598-022-12519-4)
Supplement: Supplementary file 1 — Supplementary Information. [file 41598_2022_12519_MOESM1_ESM.pdf]

**Supplementary Table 1**. Compound characteristics and vendor.			
**Compound**	***XLog P**	**Vendor (article #)**	**Structure**
Sorafenib	4.1	LC Laboratories (S-8502)	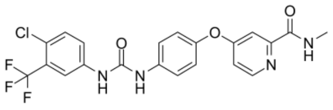
Regorafenib	4.2	LC Laboratories (R-8024)	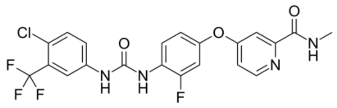
Sunitinib	2.6	LC Laboratories (S-8803)	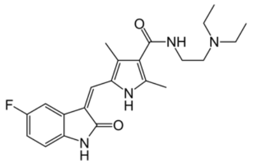
Imatinib	3.5	LC Laboratories (I-5508)	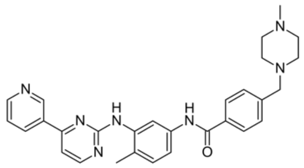
Dasatinib	3.6	LC Laboratories (D-3307)	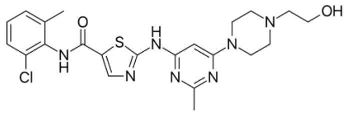
Crizotinib	3.7	Selleckchem (S1068)	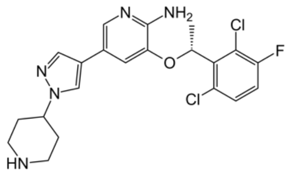
Nitazoxanide	2.0	Sigma-Aldrich (N0290)	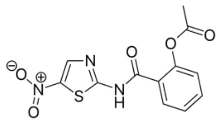
Niclosamide	4.0	Sigma-Aldrich N3510	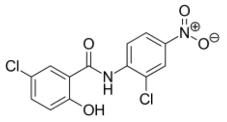
CCCP	3.4	Sigma-Aldrich (C2759)	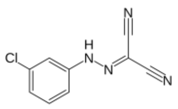
FCCP	3.7	Sigma-Aldrich (C2920)	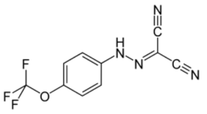
Cisplatin	NA	Accord (461201)	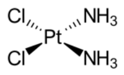

Docetaxel	1.6	Sigma-Aldrich (O1885)	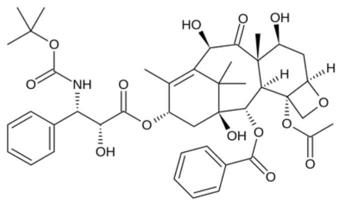
Irinotecan	3.0	Sigma-Aldrich (I1406)	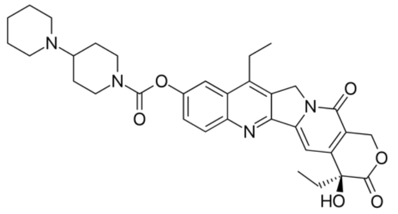
5-Fluorouracil	-0.9	Sigma-Aldrich (F6627)	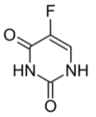
Gemcitabine	-1.5	LC Laboratories (G-4199)	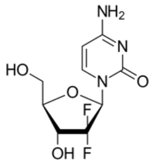

*XLogP3-AA, https://pubchem.ncbi.nlm.nih.gov, PubChem release 2019.06.1, NA = Not Available

**Supplementary Table 2**. Estimated IC_{50} values* from CRC tumoroid.	
**Compound**	**IC_{50} (µM)*<br>CRC tumoroids**
Dasatinib	89,8
Crizotinib	59,5
Sunitinib	63,1
Nitazoxanide	4,3
Sorafenib	12,8
Regorafenib	17,8

*Data analyzed using GraphPad Prism with equation log(inhibitor) vs. normalized response Variable slope (n=1, duplicate wells). IC_{50} calculations were not applicable for irinotecan, cisplatin, docetaxel, mitomycin, fluorouracil and gemcitabine.

**Supplementary Table 3**. Data analysis of raw data from tumoroid viability analysis.					
**Treatment**		**Tumoroids**			
		**x̄ difference**	**95,00% CI**	**Significance**	**P-value**
Dasatinib	90 µM	150 092	74974 - 225209	****	<0,0001
Crizotinib	90 µM	213 768	138650 - 288885	****	<0,0001
Sunitinib	90 µM	211 929	136811 - 287046	****	<0,0001
Nitazoxanide	90 µM	278 604	224108 - 333100	****	<0,0001
	30 µM	271 093	216597 - 325589	****	<0,0001
	10 µM	232 872	178376 - 287368	****	<0,0001
Sorafenib	90 µM	279 612	204495 - 354730	****	<0,0001
	30 µM	259 329	184212 - 334447	****	<0,0001
	10 µM	98 836	23719 - 173954	**	0,001
Regorafenib	90 µM	269 973	194856 - 345091	****	<0,0001
	30 µM	267 071	191953 - 342188	****	<0,0001

Data analyzed using GraphPad Prism, one way ANOVA and Dunnett´s multiple comparison test (n=1, duplicate wells). Treatment that caused viability to decrease significantly shown above.

**Supplementary Table 4**. Estimated IC_{50} values* from MCTS and monolayer viability analysis.								
**Compound**	**HCT116**				**Huh-7**			
	**MCTS**		**Monolayer**		**MCTS**		**Monolayer**	
	**IC_{50} (μM)***	**95% CI**	**IC_{50} (μM)***	**95% CI**	**IC_{50} (μM)***	**95% CI**	**IC_{50} (μM)***	**95% CI**
Nitazoxanide	1,65	1,54 - 1,77	5,40	4,95 - 5,89	1,18	1,13 - 1,22	11,34	10,80 - 11,91
Sorafenib	3,37	3,19 - 3,56	4,18	3,96 - 4,42	2,23	2,16 - 2,30	2,82	2,66 - 2,98
Regorafenib	4,51	4,22 - 4,82	3,94	3,66 - 4,25	1,62	1,53 - 1,71	1,08	1,01 - 1,16
Sunitinib	63,60	53,95 - 78,21	6,61	5,91 - 7,36	4,60	4,24 - 4,99	3,02	2,83 - 3,22
Imatinib	NA	NA	25,86	23,22 - 29,46	17,71	16,53 - 19,00	11,58	10,80 - 12,40
Irinotecan	NA	NA	6,55	5,47 - 7,93	NA	NA	5,53	4,59 - 6,73

*Data analyzed using GraphPad Prism with equation log(inhibitor) vs. normalized response Variable slope. NA = IC_{50} calculations were not applicable. HCT116: n=3 (quadruplicate wells), Huh-7: n=3 (quadruplicate wells).

Supplementary Table 5. Data analysis of raw data from HCT116 and Huh-7 MCTS viability analysis.

**Treatment**		**HCT116 MCTS**				**Huh-7 MCTS**			
		**$\bar{x}$ diff.**	**95,00% CI**	**Signif-icance**	**P-value**	**$\bar{x}$ diff.**	**95,00% CI**	**Signif-icance**	**P-value**
Irinotecan	32 µM	-5258	240760 - 282794	ns	0,999	19544	102221 - 137297	*	0,0148
	16 µM	-7099	240547 - 282581	ns	0,9985	12160	102011 - 137087	ns	0,5327
	8 µM	-4635	234559 - 276593	ns	0,9992	16327	100612 - 135688	ns	0,0962
	4 µM	-1775	224775 - 266809	ns	0,9997	21230	89899 - 124975	**	0,0047
	2 µM	494,3	171170 - 213204	ns	>0,9999	25827	71253 - 106329	***	0,0001
	1 µM	-625,4	21760 - 63794	ns	>0,9999	27304	44100 - 79176	****	<0,0001
	0,5 µM	11024	1889 - 43923	ns	0,9223	27976	27655 - 62731	****	<0,0001
Imatinib	32 µM	-10541	249927 - 291961	ns	0,9512	93144	102886 - 137962	****	<0,0001
	16 µM	-8649	238611 - 280645	ns	0,998	64999	101766 - 136842	****	<0,0001
	8 µM	-12998	230022 - 272056	ns	0,7211	39548	96539 - 131615	****	<0,0001
	4 µM	-11050	163935 - 205969	ns	0,9206	25698	81864 - 116940	***	0,0001
	2 µM	-9020	19863 - 61897	ns	0,9852	18080	46016 - 81092	*	0,0366
	1 µM	512,8	-4105 - 37929	ns	>0,9999	18381	19219 - 54295	*	0,0306
	0,5 µM	892,8	-16682 - 25353	ns	0,9999	21356	6799 - 41875	**	0,0043
Sunitinib	32 µM	88913	230650 - 272684	****	<0,0001	110486	102646 - 137722	****	<0,0001
	16 µM	55079	225509 - 267543	****	<0,0001	99929	100701 - 135777	****	<0,0001
	8 µM	27502	214557 - 256591	**	0,0014	89834	95603 - 130679	****	<0,0001
	4 µM	14017	98296 - 140330	ns	0,5881	64917	84538 - 119614	****	<0,0001
	2 µM	-3243	13705 - 55739	ns	0,9995	43177	57021 - 92097	****	<0,0001

	1 µM	-97,83	2202 - 44236	ns	>0,9999	33176	33908 - 68984	****	<0,0001
	0,5 µM	4850	-6786 - 35248	ns	0,9991	28862	19529 - 54605	****	<0,0001
Nitazoxanide	32 µM	261777	67896 - 109930	****	<0,0001	119759	92948 - 128024	****	<0,0001
	16 µM	261564	34062 - 76096	****	<0,0001	119549	82391 - 117467	****	<0,0001
	8 µM	255576	6485 - 48519	****	<0,0001	118150	72296 - 107372	****	<0,0001
	4 µM	245792	-7000 - 35034	****	<0,0001	107437	47379 - 82455	****	<0,0001
	2 µM	192187	-24260 - 17774	****	<0,0001	88791	25639 - 60715	****	<0,0001
	1 µM	42777	-21115 - 20919	****	<0,0001	61638	15638 - 50714	****	<0,0001
	0,5 µM	22906	-16167 - 25867	*	0,0198	45193	11324 - 46400	****	<0,0001
Sorafenib	32 µM	270944	-31558 - 10476	****	<0,0001	120424	75606 - 110682	****	<0,0001
	16 µM	259628	-29666 - 12368	****	<0,0001	119304	47461 - 82537	****	<0,0001
	8 µM	251039	-34015 - 8019	****	<0,0001	114077	22010 - 57086	****	<0,0001
	4 µM	184952	-32067 - 9967	****	<0,0001	99402	8160 - 43236	****	<0,0001
	2 µM	40880	-30037 - 11997	****	<0,0001	63554	541,7 - 35618	****	<0,0001
	1 µM	16912	-20504 - 21530	ns	0,2598	36757	843,0 - 35919	****	<0,0001
	0,5 µM	4336	-20124 - 21910	ns	0,9993	24337	3818 - 38894	***	0,0005
Regorafenib	32 µM	251667	-26275 - 15759	****	<0,0001	120184	2006 - 37082	****	<0,0001
	16 µM	246526	-28116 - 13918	****	<0,0001	118239	-5378 - 29698	****	<0,0001
	8 µM	235574	-25652 - 16382	****	<0,0001	113141	-1211 - 33865	****	<0,0001
	4 µM	119313	-22792 - 19242	****	<0,0001	102076	3692 - 38768	****	<0,0001
	2 µM	34722	-20523 - 21511	****	<0,0001	74559	8289 - 43365	****	<0,0001
	1 µM	23219	-21642 - 20392	*	0,0168	51446	9766 - 44842	****	<0,0001

	0,5 µM	14231	-9993 - 32041	ns	0,56	37067	10438 - 45514	****	<0,0001

Data analyzed using GraphPad Prism, one way ANOVA and Dunnett´s multiple comparison test. Treatment that caused viability to decrease significantly shown above. HCT116 and Huh-7: n=3 (quadruplicate wells).

Supplementary Table 6. Data analysis of JC-1 raw data on HCT116 and Huh-7 MCTS.

Treatment		HCT116				Huh-7			
		x̄ diff.	95,00% CI	Signif-icance	p-value	x̄ diff.	95,00% CI	Signif-icance	p-value
Nitazoxanide	32 µM	6,84	5,27 - 8,42	****	<0,0001	1,35	0,89 - 1,82	****	<0,0001
	16 µM	6,36	4,78 - 7,93	****	<0,0001	1,26	0,79 - 1,72	****	<0,0001
	8 µM	5,82	4,25 - 7,39	****	<0,0001	1,15	0,69 - 1,61	****	<0,0001
Sorafenib	32 µM	6,11	4,54 - 7,69	****	<0,0001	1,01	0,54 - 1,47	****	<0,0001
	16 µM	5,26	3,69 - 6,84	****	<0,0001	0,95	0,49 - 1,42	****	<0,0001
	8 µM	2,31	0,74 - 3,89	***	0,0007	0,62	0,16 - 1,09	**	0,0012
Regorafenib	32 µM	5,98	4,40 - 7,55	****	<0,0001	0,92	0,45 - 1,38	****	<0,0001
	16 µM	5,14	3,57 - 6,72	****	<0,0001	0,67	0,21 - 1,13	***	0,0003
	8 µM	3,50	1,92 - 5,07	****	<0,0001	0,61	0,15 - 1,08	**	0,0016
FCCP	32 µM	3,30	1,67 - 4,94	****	<0,0001	0,66	0,48 - 1,41	***	0,0004
	16 µM	5,32	3,68 - 6,95	****	<0,0001	0,84	0,38 - 1,30	****	<0,0001
	8 µM	5,44	3,87 - 7,02	****	<0,0001	0,95	0,48 - 1,41	****	<0,0001
Irinotecan	32 µM	0,63	-0,95 - 2,20	ns	0,9465	0,09	-0,38 - 0,55	ns	0,9994
	16 µM	-0,05	-1,68 - 1,59	ns	>0,9999	0,10	-0,37 - 0,56	ns	0,9993
	8 µM	0,16	-1,48 - 1,79	ns	0,9997	0,06	-0,40 - 0,52	ns	0,9996
Sunitinib	32 µM	-0,19	-1,76 - 1,39	ns	0,9996	-0,25	-0,72 - 0,21	ns	0,8593
	16 µM	-0,57	-2,21 - 1,07	ns	0,9947	-0,12	-0,59 - 0,34	ns	0,9991
	8 µM	0,12	-1,51 - 1,76	ns	0,9998	0,04	-0,42 - 0,51	ns	0,9997
Imatinib	32 µM	0,13	-1,44 - 1,71	ns	0,9997	0,15	-0,31 - 0,61	ns	0,9987
	16 µM	0,44	-1,20 - 2,08	ns	0,999	0,36	-0,10 - 0,82	ns	0,3023
	8 µM	0,52	-1,11 - 2,16	ns	0,9987	0,25	-0,21 - 0,72	ns	0,8667

Mean difference between vehicle control and treated MCTS (raw data from total fluorescence) with 95% CI of the mean difference (x̄ diff.) and adjusted p-values from one way ANOVA and Dunnett's multiple comparison test using GraphPad Prism. Ns = non-significant (p<0,05). HCT116: n=1 (quadruplicate wells) and Huh-7: n=1 (quadruplicate wells).

**Supplementary Table 7**. Statistics of JC-1 3D combination treatment on HCT116 MCTS.					
**Treatment**		**Mean Difference**	**95,00% CI**	**Significance**	**Adjusted P-Value**
Nitazoxanide	32 µM	70,85	53,64 - 88,06	****	<0,0001
	16 µM	69,16	51,95 - 86,37	****	<0,0001
	8 µM	62,35	45,14 - 79,56	****	<0,0001
	4 µM	52,32	35,11 - 69,53	****	<0,0001
	2 µM	17,84	0,6342 - 35,05	*	0,0357
	1 µM	6,966	-10,24 - 24,18	ns	0,9847
	0,5 µM	-10,04	-27,25 - 7,166	ns	0,7316
Sorafenib	32 µM	43,8	26,59 - 61,01	****	<0,0001
	16 µM	27,16	9,952 - 44,37	****	<0,0001
	8 µM	10,64	-6,572 - 27,85	ns	0,644
	4 µM	12,29	-4,917 - 29,50	ns	0,4058
	2 µM	3,953	-13,26 - 21,16	ns	0,9991
	1 µM	0,265	-16,94 - 17,47	ns	>0,9999
	0,5 µM	3,6	-13,61 - 20,81	ns	0,9992
Sorafenib 16 µM + Nitazoxanide X µM	32 µM	73,26	52,18 - 94,34	****	<0,0001
	16 µM	72,27	51,19 - 93,35	****	<0,0001
	8 µM	67,73	46,65 - 88,80	****	<0,0001
	4 µM	60,09	39,01 - 81,16	****	<0,0001
	2 µM	34,16	13,08 - 55,23	****	<0,0001
	1 µM	24,07	2,995 - 45,15	*	0,0126
	0,5 µM	6,075	-15,00 - 27,15	ns	0,9987
Sorafenib 8 µM + Nitazoxanide X µM	32 µM	74,4	53,32 - 95,48	****	<0,0001
	16 µM	71,61	50,53 - 92,68	****	<0,0001
	8 µM	67,05	45,97 - 88,12	****	<0,0001
	4 µM	63,49	42,41 - 84,56	****	<0,0001
	2 µM	28,21	7,130 - 49,28	**	0,0014
	1 µM	12,08	-8,997 - 33,16	ns	0,757
	0,5 µM	3,647	-17,43 - 24,72	ns	0,9993
Sorafenib X µM + Nitazoxanide X µM	16 µM + 16 µM	72,27	51,19 - 93,35	****	<0,0001
	8 µM + 8 µM	67,05	45,97 - 88,12	****	<0,0001

	4 µM + 4 µM	61,65	40,57 - 82,73	****	<0,0001
	2 µM + 2 µM	19,03	-2,045 - 40,11	ns	0,1134
	1 µM + 1 µM	12,36	-8,722 - 33,43	ns	0,7253
	0,5 µM + 0,5 µM	-4,26	-25,34 - 16,82	ns	0,9992

JC-1 3D assay performed on HCT116 MCTS, duplicate experiments. Mean difference between vehicle control and treated MCTS with 95% CI of the difference. Analyzed with one way ANOVA and Dunnett's multiple comparison test using GraphPad Prism. HCT116: n=2 (quadruplicate wells).

Supplementary Table 8: Clonogenic assay quantification and statistical analysis in HCT116 MCTS.

**Treatment**	**24 h**				**48 h**				**72 h**			
	**Mean**	**SD**	**Significance**	**p-value**	**Mean**	**SD**	**Significance**	**p-value**	**Mean**	**SD**	**Significance**	**p-value**
Nitazoxanide 8 µM	187,6	18,91	ns	0,3992	25	15,29	****	<0,0001	0,8889	2,315	****	<0,0001
Nitazoxanide 4 µM	200	0	ns	>0,9999	122,7	67,78	****	<0,0001	34,11	54,25	****	<0,0001
Nitazoxanide 2 µM	200	0	ns	>0,9999	200	0	ns	>0,9999	196	20,22	ns	0,9996
Sorafenib 16 µM	121,1	20,92	****	<0,0001	17	4,093	****	<0,0001	0,2222	0,667	****	<0,0001
Sorafenib 8 µM	189,4	18,06	ns	0,5869	41,56	12,32	****	<0,0001	3,556	2,92	****	<0,0001
Sorafenib 4 µM	200	0	ns	>0,9999	194,7	26,8	ns	0,9994	53	51,4	****	<0,0001
Irinotecan 8 µM	200	0	ns	>0,9999	193,6	11,98	ns	0,9972	183,9	25,11	ns	0,7534
Imatinib 8 µM	200	0	ns	>0,9999	200	0	ns	>0,9999	200	0	ns	>0,9999
Nitazoxanide 4 µM + Sorafenib 8 µM	127,5	22,01	****	<0,0001	14	7,563	****	<0,0001	0	0	****	<0,0001
Nitazoxanide 2 µM + Sorafenib 4 µM	185,5	16,53	ns	0,2754	36,83	26,22	****	<0,0001	1,833	2,714	****	<0,0001

Maximum number of clones were set to 200, One way ANOVA and Dunnett’s multiple comparisons test. For nitazoxanide, sorafenib and irinotecan n= 3 (triplicate wells). For combinations and imatinib n=2 (triplicate wells).

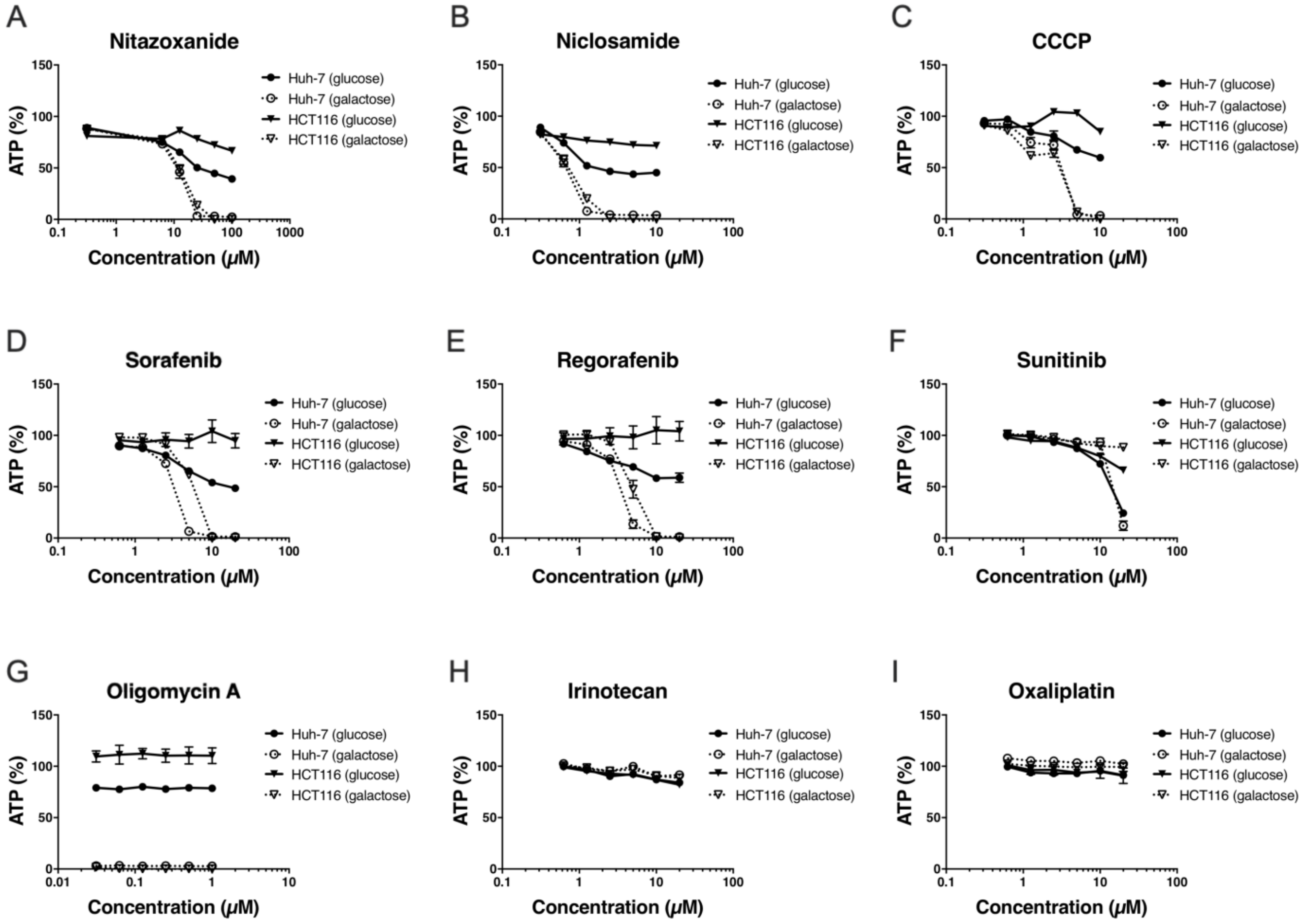

Supplementary Figure 1. ATP measurements after treatment of cells lines grown in glucose or galactose containing media. HCT116-GFP cells (triangles) and Huh-7 cells (circles) were grown in glucose (closed symbols) and galactose (open symbols) and incubated in the presence of nitazoxanide (A), niclosamide (B), CCCP (C), sorafenib (D), regorafenib (E), sunitinib (F), oligomycin A (G), irinotecan (H) and oxaliplatin (I) at the doses indicated. After 24 hours treatment, the ATP content was measured. Data are expressed as mean ±SD, n= 3 (triplicate wells). In some cases the error bars are shorter than the size of the symbol and expressed as percent of vehicle control (DMSO).

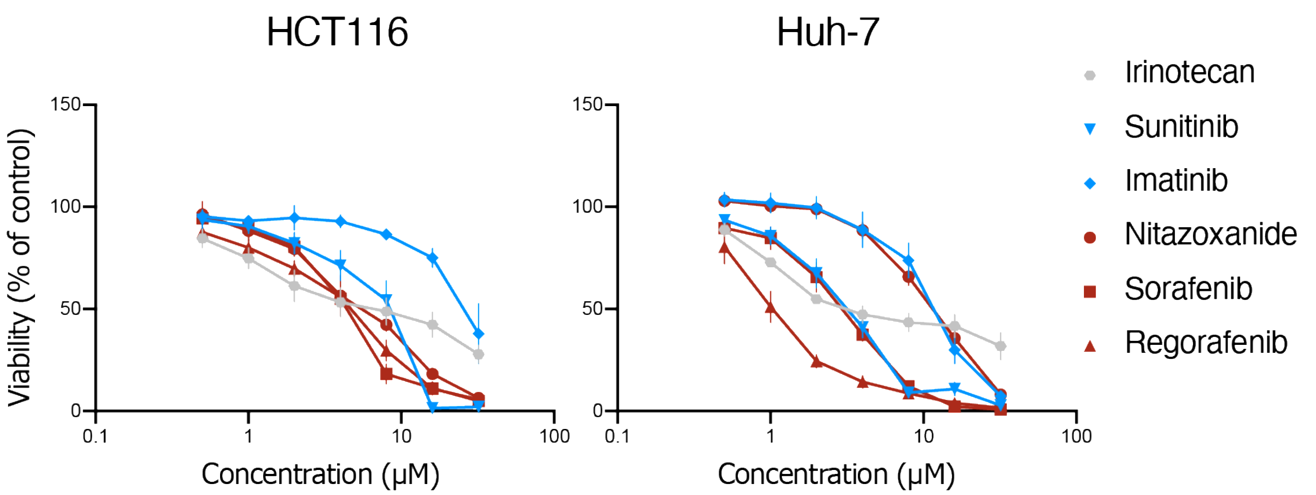

Supplementary Figure 2. Treatment response for cells in 2D. Viability, after treatment with six compounds for 72 h, measured using ATP-assay (plotted as mean ± SD). HCT116: n=3 (quadruplicate wells), Huh-7: n=3 (quadruplicate wells).

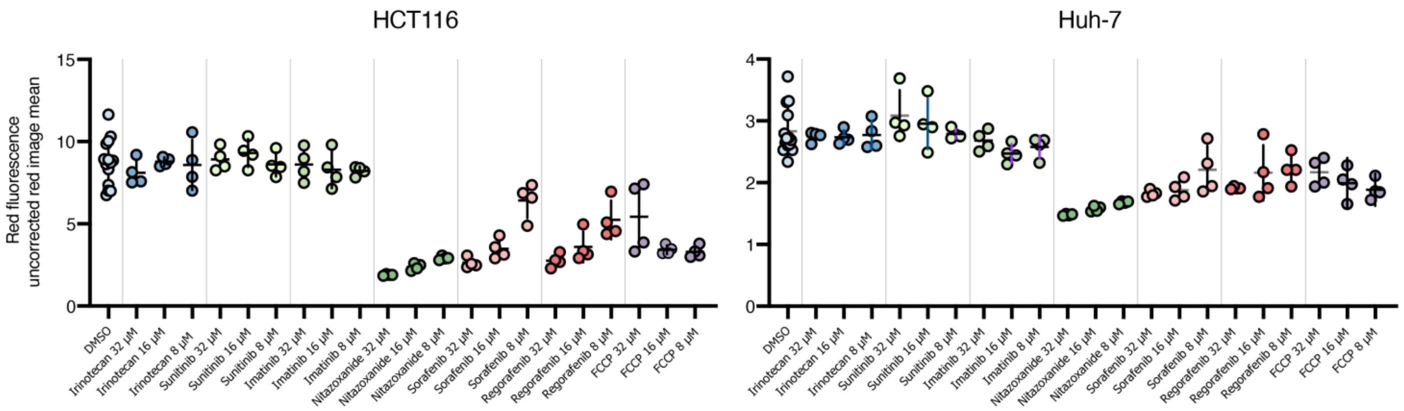

Supplementary Figure 3. JC-1 raw data. Data from one representative experiment. Plotted as mean with range.

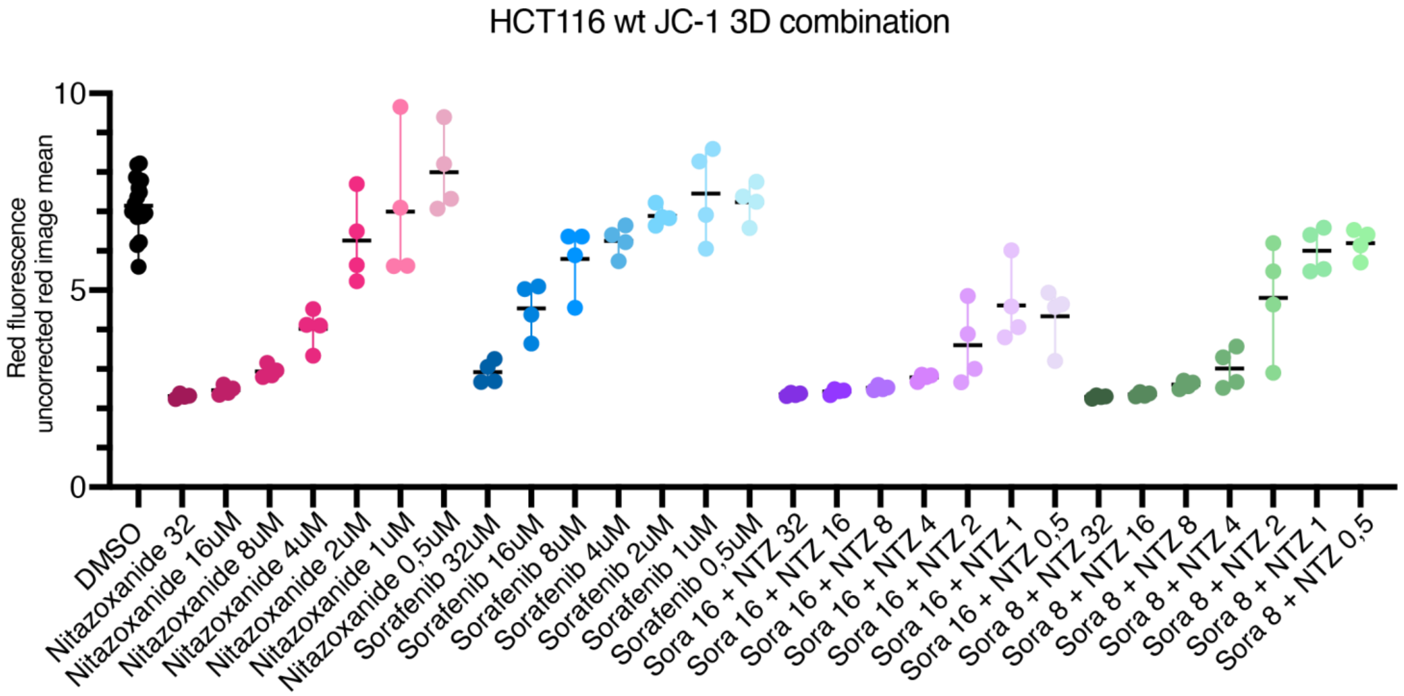

Supplementary Figure 4. Combining sorafenib and nitazoxanide generates additive effects in HCT116 MCTS. JC-1 raw data. HCT116: n=2 (quadruplicate wells). Plotted as mean with range.

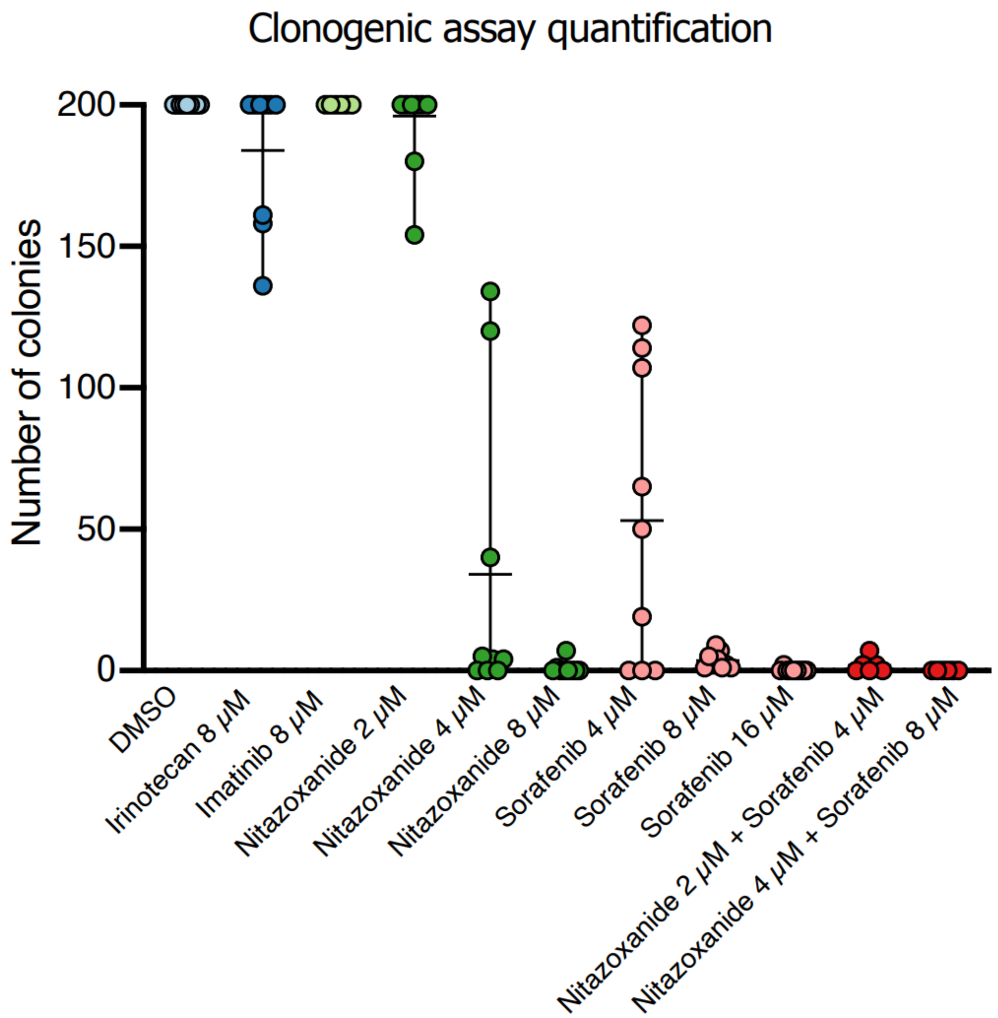

Supplementary Figure 5. Clonogenic assay quantification after 72 h of treatment. For nitazoxanide, sorafenib and irinotecan n= 3 (triplicate wells). For combinations and imatinib n=2 (triplicate wells). Plotted as mean with range.
